# Additive Manufacturing of Transparent Multi‐Component Nanoporous Glasses

**DOI:** 10.1002/advs.202305775

**Published:** 2023-10-23

**Authors:** Beining Li, Zhenjiang Li, Ido Cooperstein, Wenze Shan, Shuaipeng Wang, Benxue Jiang, Long Zhang, Shlomo Magdassi, Jin He

**Affiliations:** ^1^ Key Laboratory of Materials for High Power Lasers Shanghai Institute of Optics and Fine Mechanics Chinese Academy of Sciences Shanghai 201800 China; ^2^ College of Materials Science and Opto‐Electronic Technology University of Chinese Academy of Sciences Beijing 100083 China; ^3^ Shanghai Institute of Applied Physics Chinese Academy of Sciences Shanghai 201800 China; ^4^ Casali Center of Applied Chemistry Institute of Chemistry The Hebrew University of Jerusalem Jerusalem 9190401 Israel

**Keywords:** DLP, hybrid oligomer ink, hydrophobic modification, multi‐component, photoluminescence, transparent nanoporous glasses

## Abstract

Fabrication of glass with complex geocd the low resolution of particle‐based or fused glass technologies. Herein, a high‐resolution 3D printing of transparent nanoporous glass is presented, by the combination of transparent photo‐curable sol–gel printing compositions and digital light processing (DLP) technology. Multi‐component glass, including binary (Al_2_O_3_‐SiO_2_), ternary (ZnO‐Al_2_O_3_‐SiO_2_, TiO_2_‐Al_2_O_3_‐SiO_2_), and quaternary oxide (CaO‐P_2_O_5_‐Al_2_O_3_‐SiO_2_) nanoporous glass objects with complex shapes, high spatial resolutions, and multi‐oxide chemical compositions are fabricated, by DLP printing and subsequent sintering process. The uniform nanopores of Al_2_O_3_‐SiO_2_‐based nanoporous glasses with the diameter (≈6.04 nm), which is much smaller than the visible light wavelength, result in high transmittance (>95%) at the visible range. The high surface area of printed glass objectives allows post‐functionalization via the adsorption of functional guest molecules. The photoluminescence and hydrophobic modification of 3D printed glass objectives are successfully demonstrated. This work extends the scope of 3D printing to transparent nanoporous glasses with complex geometry and facile functionalization, making them available for a wide range of applications.

## Graphical abstract

This work reports metal chelates sol–gel route to 3D print complex‐shaped macroscopic transparent glass structures with inherent porosity and broad compositional design space. This method extends the transparent glass additive manufacturing technology from classical dense glass to the field of nanoporous multi‐component glass, bearing significant promise for their utilization in broad applications, including in light‐emitting devices and sensors.

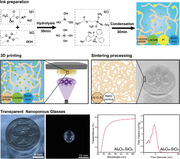

## 1 Introduction

Glass is one of the fundamental materials in the history of human civilization dating back to Egyptian or Mesopotamia times. It has been widely used in optics, architecture, medicine and art owing to its unique properties such as optical transparency, high thermal and chemical stability, rapid manufacturing process, superior shaping ability, etc.^[^ ^1^, ^2^, ^3^ ^]^ In traditional glass manufacturing, two main forming processes, blowing and casting which were invented in ancient times, were widely adopted. The innovations in glass manufacturing since the Industrial Revolution played a pivotal role in creating new glass products, which lead to several important high‐tech revolutions. For example, the float glass process enables the manufacture of large flat windows. The fusion‐draw process makes possible high‐strength ultra‐thin glass for smartphones. The continuous filament process for manufacturing glass fibers opened the era of optical communication.

Additive manufacturing (3D printing) is a revolutionary technology in the manufacturing field, which opened new possibilities for manufacturing of complex‐shaped objects in the fully digital design process, thus arousing the increasing interest worldwide. In the context of glass, several 3D printing breakthroughs have been achieved to fabricate complex shape glass objects covering several orders of magnitude from meter to ≈100 nm. For instance, directly extruding quartz or chalcogenide glass in a molten state,^[^ ^4^, ^5^ ^]^ the vat photopolymerization (Digital Light Process, DLP or Stereolithography) of the glass slurry^[^ ^6^ ^]^ or sol–gel precursors,^[^ ^7^ ^]^ selective laser melting (SLM) using glass powders,^[^ ^8^ ^]^ direct ink writing (DIW) with glass slurry.^[^ ^9^, ^10^, ^11^, ^12^ ^]^ Recently, 3D printing technology based on ultrafast laser direct writing combined with sintering processing has achieved the fabrication of dense glass micro‐nanostructures,^[^ ^13^, ^14^, ^15^ ^]^ especially 3D printing of silica glass at nanoscale was reported via Two‐Photon Polymerization (TPP).^[^ ^16^, ^17^ ^]^ Extending the chemical composition is another major pursuit in glass additive manufacturing, high optical quality TiO_2_‐SiO_2_,^[^ ^9^ ^]^ GeO_2_‐SiO_2_,^[^ ^10^ ^]^ GeO_2_‐TiO_2_‐SiO_2_^[^ ^11^ ^]^ glasses 3D printing was achieved by combining sol–gel ink, DIW and sintering. Multicomponent art glass bottles were 3D printed based on high‐temperature molten glass using multiple nozzles.^[^ ^18^ ^]^ TiO_2_‐SiO_2_ glasses with compositional gradients were 3D printed using an upgraded direct ink writing technology with an active inline micromixer.^[^ ^12^ ^]^ SiO_2_‐B_2_O_3_‐P_2_O_5_ glass parts^[^ ^19^ ^]^ and Er^3+^/Yb^3+^ co‐doped SiO_2_‐P_2_O_5_ glass^[^ ^20^ ^]^ are DLP 3D printed by combining the hydrolysis of metal alkoxide. SLM has also been used to 3D printing the multi‐component glass such as Li_2_O‐Al_2_O_3_‐SiO_2_, based on the glass powder.^[^ ^21^ ^]^ However, there are still many challenges in 3D printing of glass including the limited printable glass systems, complex and time‐consuming printing process, limited precision, and relatively low optical quality. Till now, the printing glass systems are mainly limited to silica glass systems,^[^ ^6^, ^19^, ^22^, ^23^ ^]^ and dense non‐porous glasses.^[^ ^9^, ^10^, ^11^, ^12^, ^13^, ^14^ ^]^ Therefore, broadening the glass types and compositional design space with high printing resolution is key in advancing the 3D printing glass technologies.

Transparent nanoporous glass (NPG) is a promising optical material, which inherits the excellent physical and chemical stability of inorganic glass with a high specific surface area and pore structure. Due to its unique properties, the NPG has found wide applications in the manufacture of nanofilters,^[^ ^24^ ^]^ substrates of biological preparations, nano‐confined optical matrix for luminescent nanocrystals or rare earth ions,^[^ ^25^, ^26^ ^]^ photonic crystal fiber laser^[^ ^27^ ^]^ and the production of micro‐optic components.^[^ ^28^ ^]^ The most typical NPG, which is well known as Vycor glass commercialized by Corning company, was fabricated using spinodal decomposition in multi‐component oxide glass and leaching out one of the decomposed phases with the acid solution.^[^ ^29^ ^]^ However, heat treated phase separation technology is not compatible with 3D printing technologies due to the tedious post treatment process.^[^ ^29^, ^30^ ^]^ Therefore, direct printing of a transparent NPG is currently out of reach. Sol–gel process, which inherently creates nanopores via the hydrolysis and condensation reactions of metal salts, offers a distinct pathway to fabricate NPG with high affinity to 3D printing technology. The sol–gel process results in particle‐free transparent composition, which significantly benefits the photopolymerization process. Recently, the combination of the sol–gel process and photo‐cured 3D printing technology led to successfully fabricated objects with high geometrical complexity in several vitreous materials systems, such as ordered mesoporous silica,^[^ ^31^ ^]^ silica glass,^[^ ^6^ ^]^ bioactive glass^[^ ^32^ ^]^ and ceramic aerogels.^[^ ^33^ ^]^ Although the ordered mesoporous silica composite structure can be realized by preparing the mixed precursor ink through the templating agent,^[^ ^31^ ^]^ this structure is opaque and has low printing accuracy, so it is not suitable for optics and photonic device applications. In some studies, the porosity in the final or intermediate glass product was revealed.^[^ ^19^, ^29^, ^33^, ^34^, ^35^ ^]^ For example, photoinduced phase separation precursor inks can also achieve a porous structure,^[^ ^19^ ^]^ but it only exists in the intermediate product, and the final glass structure still needs to be densified at high temperature. Morover, such products generally lack transparency owing to the existence of large pores which scatter the light significantly, limiting their applications in optics.

In this study, we report the advantages of 3D printing via digital light processing (DLP) and sol–gel process, which enables the formation of macroscopic 3D transparent glass objects with inherent uniform porosity and multi‐oxide chemical compositions. Recently, we and collaborators developed a template‐free sol–gel route using metal chelates, which results in transparent nanoporous fully polymerized glasses in several compositional systems including AlPO_4_,^[^ ^36^ ^]^ GaAlPO_4_,^[^ ^37^ ^]^ AlPO_4_‐SiO_2_,^[^ ^38^ ^]^ and Al_2_O_3_‐SiO_2_.^[^ ^30^ ^]^ In this sol‐gel route, organic chelating ligand leads to a gel with 3D continuous structure of the preceramic metal‐organic polymer, which is pyrolyzed to form a transparent NPG.

Herein, we exploit metal chelates sol‐gel route to 3D print complex‐shaped macroscopic transparent glass structures with inherent porosity and broad compositional design space. Compared to their non‐porous counterparts, nanoporous glasses offer great advantages for designing functional devices via the adsorption and loading of target molecules and ions.^[^ ^39^, ^40^, ^41^, ^42^ ^]^ As proof of concept, we produced superhydrophobic and photoluminescence devices by loading functional molecules into nanopores. This work extends the glass additive manufacturing technology from classical dense glass to the field of nanoporous multi‐component glass, bearing significant promise for their utilization in broad applications, including in light‐emitting devices, photovoltaics, and sensors. The developed sol‐gel ink would also have significant potential in combining with femtosecond laser‐based 3D printing technology (a reliable tool for additive manufacturing of nanoscale devices, such as TPP) for micro‐nano NPG device fabrication. ^[^ ^39^ ^]^

## 2 Results and Discussion

### 2.1 Working Principle

**Figure 1:**
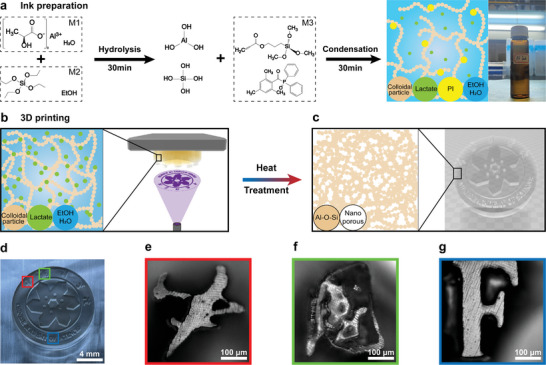
a) Fabrication process of nanoporous aluminosilicate glass. Aluminum L‐lactate, APTMS, TPO, and TEOS for synthesis. b) UV curable hybrid oligomer ink by sol–gel method, schematics of the DLP, illustrating the geometrical complexity generated by the illumination, the 3D printed logo of the Chinese Academy of Sciences (gel), and c) transparent nanoporous glass logo structure (CAD mold) after the sintering process. d) 3D printed logo of the Chinese Academy of Science nanoporous glass after heat treatment and some details of microscopic observation in which the red, green, and blue boxes point to e), f), and g) respectively.

The fabrication of 3D complex‐shaped transparent NPG objects is based on the formation of new photo‐curable sol–gel printing composition (“ink”), DLP printing, aging and calcination, as schematically shown in **Figure** **1**. To illustrate the working principle, we developed a photo‐curable sol–gel ink composed of silica precursor, aluminum lactate, metal salts and photo‐polymerizable monomers (Figure 1a). Aluminum lactate acts as the molecular precursors of alumina and pore‐forming agents simultaneously. When the silica precursor, tetraethyl orthosilicate (TEOS), is added to the aluminum lactate aqueous solution, a sol–gel process starts to form a silica‐alumina network, by hydrolysis and condensation reactions. In this stage, other metal salts can be added as well to form silica‐alumina based multi‐component glass. For enabling the photopolymerization process by the DLP printing, the above solution is mixed with a UV‐curable monomer composition that consists of UV‐curing monomer APTMS and photo‐initiator TPO. We select a silane coupling agent (APTMS) as the UV‐curing monomer which will participate in the formation of the Al‐O‐Si glass network at the photopolymerization stage, thus significantly enhancing the structural integrity of the glass network.

To enable the high‐quality DLP printing, low‐viscosity liquid ink is required. It is crucial to keep the sol state of ink during printing, which is achieved by adjusting the pH (3‐4)^[^ ^43^ ^]^ using the HNO_3_, and the water to TEOS ratios according to TEOS‐H_2_O‐EtOH ternary phase diagram.^[^ ^44^ ^]^ After the solution turns clear, it continues to be stirred for 30 min and subsequently transferred to the 3D printing vat. The ink consisted with clear yellowish oligomer was printed by a commercial DLP printer (Figure 1b), where the 3D object was fabricated in a layer‐by‐layer manner. In this process, each layer is irradiated by patterned UV light for a predefined time, to initiate a local photopolymerization of the acrylic groups in the oligomers. After printing, a 3D structured object (Demo: logo of Chinese Academy of Science, Figure 1b right) in wet gel state is obtained. The uniform distribution of lactate in an extended three‐dimensional gel network would play a vital role in the formation of uniform nanopores in the calcination stage (Figure 1b left). More 3D printed gel objects with high geometrical complexity are presented in Figure S1 (Supporting Information). The centimeter‐level triply periodic minimal surface structure and the letter “SIOM” (Figure S2, Supporting Information), and the millimeter‐level structure such as photonic gate and lattice (Figure S3, Supporting Information) of transparent nanoporous glasses after sintering.

The microporous structure in the printed gel is formed during the polycondensation of precursors. By aging and calcination, the microporous will be aggregated into larger mesopores via the removal of organic matter, which includes three steps: 1) the wet gel structure was aged and dried at 70 °C for 7 days to remove solvents and enhance the strength of gel, 2) nanoporosity was formed by the thermal decomposition of the lactate at 400 °C for 120 min, 3) the final glass object was obtained by sintering at 700 °C for 2 h to complete the decomposition of organic ligands (Figure 1c). During the formation of the glass structure, Al^3+^ compensate for the charges of three non‐hybridized oxygen atoms generated by the rupture of the 3/2 Si‐O‐Si connection, resulting in a tetramer composed of one Al(VI) unit and three Al(IV) units.^[^ ^38^, ^45^ ^]^ This is a reconstruction of the charge‐balanced Al^3+^(AlO_4_^−^)_3_ unit. Nonbridging oxygen atoms on Si atoms can be linked with Al. The silica and alumina networks penetrate each other after condensation and resulting that the metal salt enters the glass network to form Al_2_O_3_‐SiO_2_ glass. The resulting objects are transparent glass monoliths with high geometrical complexity. Typical printed complex‐shaped 3D nanoporous glass structures (logo of the Chinese Academy of Sciences) after removal of all the organic material are presented in Figure 1d, the boundary and structure of the printed object are visible, and some details of microstructure are shown in Figure 1e–g.

### 2.2 The Formation of Nanopores During Gel‐Glass Transition

The formation of uniform nanopores is important for obtaining transparent nanoporous glasses, opening many applications such as super hydrophobicity^[^ ^46^ ^]^ and nano‐confined functionalization. One advantage of the presented method lies in creating nanopores via the template‐free metal chelate sol–gel process, in which the organic chelate ligands act as the pore‐creating agents.

**Figure 2:**
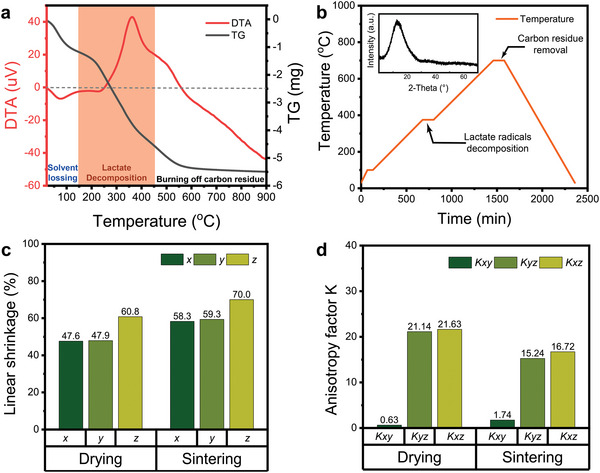
a) Characterization of the heat treatment of 3D printed monolith. TG/DTA analysis results include the process of solvent losing, lactate decomposition (orange range), and burning off organic matter. b) Lactate is fully decomposed to form mesopores and remove carbon residue, c) the illustration is XRD pattern of NPG. d) Linear shrinkage and shrinkage anisotropy factor *K* of the drying and sintering stages.

Uniform nanoporous structures and the transition of gel‐glass were accomplished in this work by heat‐treating the 3D printed objects. This heat treatment includes three steps: 1) aging and drying, 2) chelating ligands decomposition, 3) burning out of carbon residues. The aging and drying process is carried out in an oven at 70 °C for 7 days, which effectively removes the water and ethanol from the wet gel. The condensation reaction continued during the aging process to strengthen the glass network,^[^ ^44^, ^46^, ^47^, ^48^ ^]^ resulting in xerogel objects with complex structures. Thermal analysis (TG/DTA) of samples can provide guidance for setting the post‐printing heat treatment process. The weight and thermal changes of the printed green body with temperatures are analyzed by Thermogravimetry‐differential thermal analysis (TG‐DTA), as shown in **Figure** **2**a. It presents three steps in the thermal gradient: 1) Solvent loss (50–150 °C), 2) Lactate radical decomposition (150–450 °C), 3) Burning off carbon residue (450–600 °C). The weight loss at each step is 19.01%, 15.41%, and 6.60%, respectively. In step 1, the DTA curve shows a small endotherm peak around 100 °C, corresponding to water evaporation. In step 2, the exothermic peak around 361 °C can be attributed to the decomposition of lactate ligands. To ensure the complete decomposition of lactate moieties, it is chosen to keep it at 400 °C for 120 min (Figure 2b). In step 3, a small exothermic peak at 500 °C is associated with the formation of carbonaceous compounds.^[^ ^19^ ^]^ Sol–gel process is a wet chemistry route which formed the glass network at low temperature. According to nuclear magnetic resonance results, inorganic glass network can be obtained by sintering at around 650 °C.^[^ ^45^, ^49^ ^]^ The glass transition temperatures (*T*_g_) of the four components (Al_2_O_3_‐SiO_2_, TiO_2_‐Al_2_O_3_‐SiO_2_, ZnO‐Al_2_O_3_‐SiO_2_, and CaO‐P_2_O_5_‐Al_2_O_3_‐SiO_2_) were characterized by DSC, and the *T*_g_ of all samples exceeded 800 °C (Figure S4, Supporting Information). Sintering at high temperatures (>*T*_g_) enable the viscous flow of glass structure, which leads to the collapse of nanoporous structure.^[^ ^50^ ^]^ In order to maintain the unique nanoporous structure in pure transparent glass, the 3D printing object was sintered at 700 °C for 120 min to completely remove carbon residues, as shown in Figure 2b.

In addition, to prove that the gel indeed formed a glass after sintering, the samples were characterized by XRD as shown in the inset of Figure 2b in which the sharp diffraction peak is absent, indicating that the obtained object is fully polymerized glass. FTIR spectroscopy also shows that as the temperature increases, the infrared characteristic peaks of organic matter in the xerogel gradually weaken and then disappear^[^ ^51^, ^52^ ^]^ as shown in Figure S5 (Supporting Information), which indicates that the heat‐treated sample no longer contains carbonaceous compounds and achieves the preparation of inorganic glass.

Due to the size difference between the final sintered nano‐porous glass and printed gel as a result of the removal of organic matter in the printed objects, it is important to track the shrinkage of transparent NPGs parts in each step. The linear shrinkage (*S*) of the 3D printed objects after drying and sintering is anisotropic, as measured by dimensional changes in *x, y, z*‐axes of a heat‐treated object compared to the printed object, which was summarized in **Table** **1**. Therefore, the anisotropy factor (*K*) can be introduced to evaluate its influence on the final dimensional precision of the NPG. The linear shrinkage is similar in the *x* and *y* direction but presents the obvious anisotropy in *z* direction (Figure 2c). The anisotropy factor *K_xy_* (0.63, 1.74) was small, and the anisotropy factors (*K*_yz_ = 21.14, 15.24; *K*_xz_ = 21.63, 16.72) significantly increased (Figure 2d). The reason for this difference is that the DLP is a top‐down, surface‐to‐volume printing process, in which the printing in the *x* and *y* directions formed by the 2D pattern was parallel to the platform. Therefore, it led to similar linear shrinkage values for *S*_x_ and *S*_y_, and a small anisotropy factor *K*_xy_. Instead, DLP overexposes subsequent layers by prolonging the exposure time so that they would interconnect with the previous layer in *z* direction. These shrinkage results prove that the 3D printed transparent NPGs does not distort the geometry of the original design, providing reference information for device design.

**Table 1:** Sample size dimensions (mm) in the *x*,*y*,*z* directions for the 3D printing, aging and drying, sintering stages.

	3D printing [mm]	Aging and drying [mm]	Sintering [mm]
*x*	10	5.24	4.17
*y*	15	7.81	6.10
*z*	3	1.18	0.90

**Figure 3:**
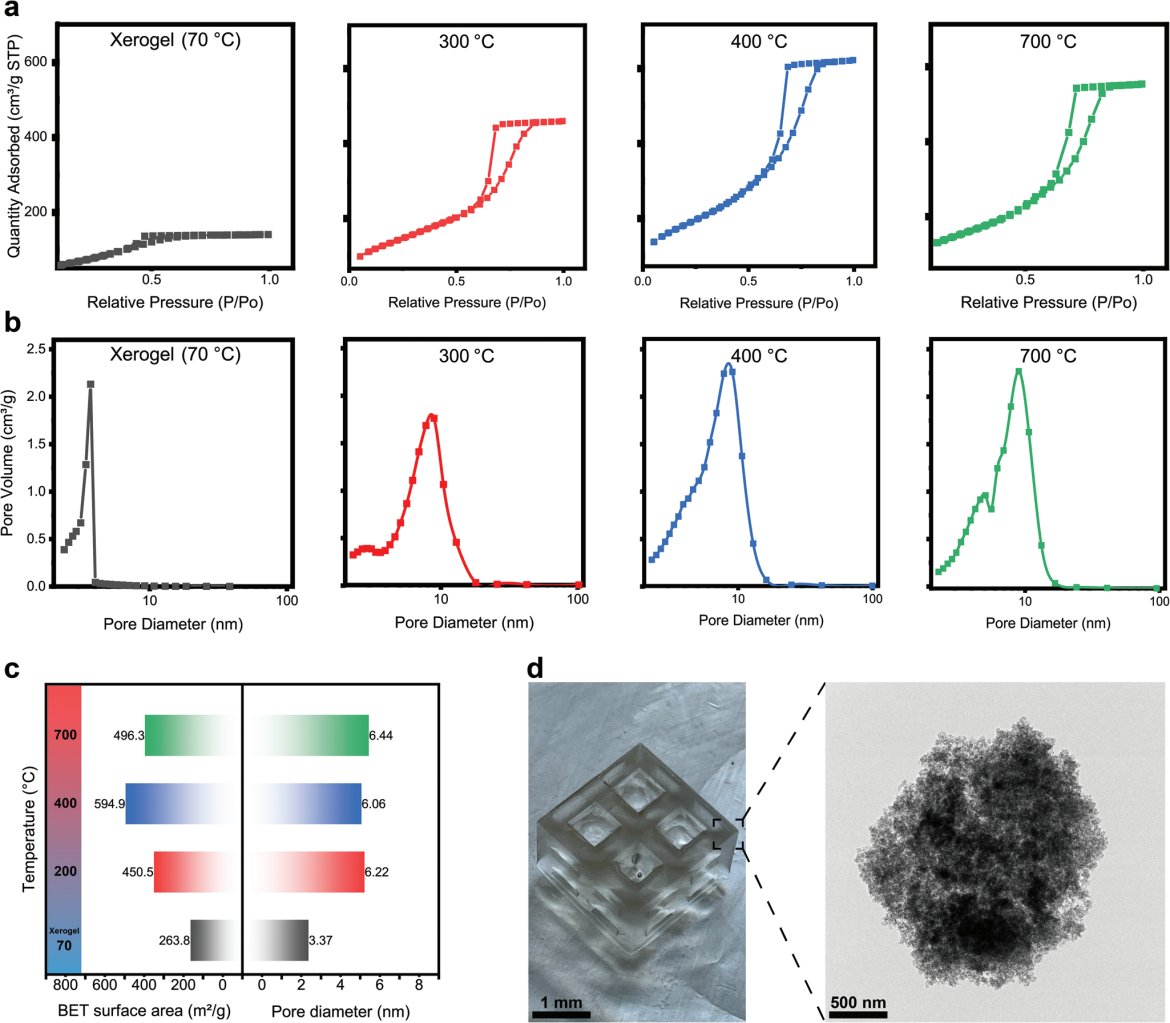
a) Characterization of the nanopores structure in 3D printed glass. BET Isotherm at dry gel (black), 300 °C (red), 400 °C (blue), 700 °C (green). b) Pore volume distribution of the dry gel (black), 300 °C (red), 400 °C (blue), and 700 °C (green) samples. c) Variation of BET surface area and pore diameter with temperature. d) The nanoporous glass structure and TEM image of the sample sintered at 700 °C.

BET and TEM measurements were performed to probe the nanoporous structure evolution in the heat treatment process. The specific surface area, pore diameter, BET isotherm, and pore diameter volume distribution of NPG at each sintering stage are shown in **Figure** **3** to reveal the evolution of the pore structure. The surface area was calculated from the BET isotherm report as shown in Figure 3a, the curves of different colors represent the heat treatment temperatures of dry gel (70 °C, black), 300 °C (red), 400 °C (blue), and 700 °C (green), respectively. The pore volume distribution of the dry gel (black), 300 °C (red), 400 °C (blue), and 700 °C (green) sample was analyzed by Barrett−Joyner−Halenda (BJH) desorption as shown in Figure 3b. The N_2_ adsorption and desorption curves at different temperatures were all typical type IV curves according to the IUPAC classification, indicating the micropores of the wet gel already merged into mesopores in dry gel with the evaporation of water and ethanol. With the increase of temperature from 70 °C (dry gel) to 300 °C, the surface area significantly increased from 263.8 to 450.4 m^2^ g^−1^ and pore size was expanded to 6.2 nm. As revealed in the DTA data, the evaporation of solvent and particularly the onset of decomposition of the lactic acid group leads to the formation of uniform nanopores in glasses. With the temperature increasing to 400 °C, the decomposition of lactate moieties was almost completed, thus resulting in the peak values for the specific surface area (594.9 m^2^ g^−1^) and pore volume of the sample. However, as the temperature increases from 400 to 700 °C, the surface area decreases slightly to 496.3 m^2^ g^−1^ (Figure 3c). This can be attributed to the densification trend of high surface area nanoporous glass driven by the high surface energy. TEM image of the NPG sintered at 700 °C (Figure 3d) showing a spongy nanoporous structure, which further indicates the successful 3D printing of NPG. The same result as shown in Figure S6 (Supporting Information) is also observed in the glass BET data of other components (TiO_2_‐Al_2_O_3_‐SiO_2_, ZnO‐Al_2_O_3_‐SiO_2_, and CaO‐P_2_O_5_‐Al_2_O_3_‐SiO_2_), the pore size distribution is concentrated around 10 nm, and the specific surface area is higher than 450 m^2^ g^−1^. The uniform nanopores of the NPG mainly arise from the thermal decomposition of lactate moieties, leading to the unique combination of high transparency and high surface area in 3D‐printed NPG.

### 2.3 High Resolution 3D Printing High Complex‐Shaped and Multi‐Component Nanoporous Glass

The fabrication of complex‐shaped multi‐component NPG objects with high accuracy by vat photopolymerization was achieved in this work. The high transparency of the sol–gel ink enables printing complex structures on the micron scale, due to the highly transparent ink effectively preventing the scattering of ultraviolet light at the edge of the print, thus enabling the high consistency between the projection pattern and the curing pattern. In this work, the objects were printed with a combination of transparent sol–gel ink and a commercial DLP printer. DLP printing technology offers high resolution, which can create very fine details and intricate geometries with precision.^[^ ^53^ ^]^ Moreover, it produces very little distortion or warping of the printed parts, as the layers are cured quickly and evenly.

**Figure 4:**
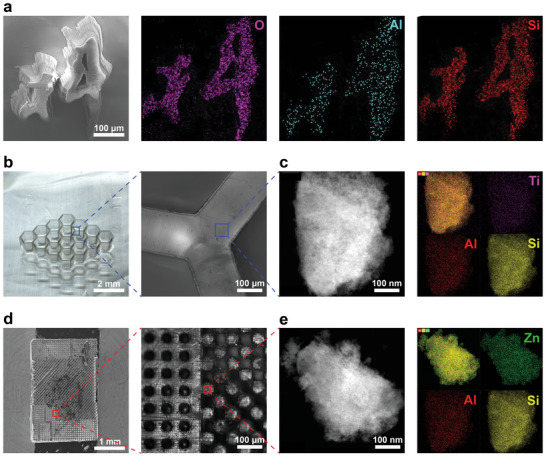
a) The SEM image of Chinese character, EDS elemental mapping for O, Al, Si showing the composition of the part of 3D printed nanoporous glass Chinese Academy of Science logo. b) Hexagonal honeycomb samples and detail displays, c) its TEM image and EDS elemental mapping for Ti, Al, Si; d) SEM image of 3D printed of micron‐scale devices, column arrays and hole array aggregates, e) its TEM image and EDS elemental mapping for Zn, Al, Si.

The high‐resolution, complex‐shaped characteristics of the printed NPG after sintering process are demonstrated in the SEM images of Chinese characters with several hundred microns (**Figure** **4**a) and a mechanically stable honeycomb structure in which the joints of the objects are smooth and orderly, with an edge length of ≈200 µm (Figure 4b). The linear shrinkage of 3D printed objects is about 70%, which further enables the fabrication of sub‐printing resolution micro‐scale devices. Furthermore, we were able to create 3D objects with spatially controlled nanoporous structures. A photonic gate is a device that controls the flow of photos (light particles).^[^ ^54^ ^]^ Photonic gates are important components in optical communication systems, as they enable the routing and processing of optical signals. It is used in various applications, such as in optical computing, quantum information processing, and fiber‐optic communication networks.^[^ ^54^, ^55^, ^56^ ^]^ A photonic gate (3.5 × 6 × 3 mm) model with column arrays and hole array had been designed and printed, the size of a single column and hole is about 50 µm after heat treatment (Figure 4d). The flexibility to create the complex‐shaped 3D NPG objects with high resolution grants various potential optical applications.

The sol–gel process offers a great advantage for introducing a variety of components, such as metal salts, during the hydrolysis of metal alkoxides, enabling making multi‐component printed glass. It is demonstrated by energy dispersive X‐Ray spectroscopy (EDS) elemental mapping as shown in Figure 4. Al, O, and Si in Al_2_O_3_‐SiO_2_ NPG are evenly distributed on micro‐Chinese characters (Figure 4a). Furthermore, ternary component NPG TiO_2_‐Al_2_O_3_‐SiO_2_ and ZnO‐Al_2_O_3_‐SiO_2_, and quaternary‐component CaO‐P_2_O_5_‐Al_2_O_3_‐SiO_2_ glass objects were successfully printed by adding TBT, ZnAc, and L‐Ca and NH_4_H_2_PO_4_ to the Al_2_O_3_‐SiO_2_ sol–gel ink system. The elemental distribution in the multi‐component glass was analyzed using a transmission electron microscope (TEM) and EDS, respectively. We have successfully achieved 3D printing NPG from binary glass to ternary and quaternary glasses, while Ti and Zn are uniformly distributed (TEM images and mapping of TiO_2_‐Al_2_O_3_‐SiO_2_ NPG and ZnO‐Al_2_O_3_‐SiO_2_ NPG are shown in Figure 4c,e respectively). The slightly segregation of Ca^2+^ occurs in the CaO‐P_2_O_5_‐Al_2_O_3_‐SiO_2_ NPG as shown in Figure S7 (Supporting Information). This is probably due to CaO is not a glass network former but classified as network modifier, which is integrated into glass network with the help of the heat treatment process.^[^ ^57^ ^]^ It is worth mentioning that the introduction of P and Ca elements during the preparation of ink by the sol–gel method did not cause ink turbidity and phase separation, indicating that the preparation of multi‐component UV‐curable ink was successful. This work successfully extended UV‐curable sol–gel ink from silica glass to binary, ternary even quaternary glass systems, which represents an important step forward in yielding the chemical versatility and porosity to 3D printing glass technology.

### 2.4 Demo of Photonic Functionalized 3D Nanoporous Glass Object

Uniform nanoporous structures of NPG offer great possibilities for post‐functionalization in post 3D printing process due to their high specific surface area and chemical diversity. The high transparency of NPG is highly promising for optical applications.

**Figure 5:**
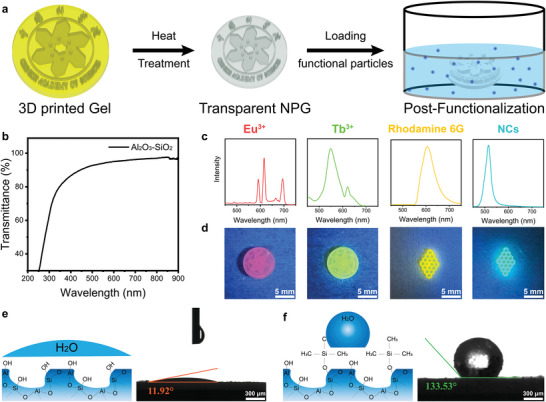
a) The post‐functional application of NPG. Schematic diagram of the postprocessing strategy for the loading method. b) UV–vis optical transmission spectra of nanoporous glasses. c,d) Photoluminescence emission of Rare Earth Ions (Eu^3+^, Tb^3+^), Laser Dye (Rhodamine 6G) and Luminescent Nanocrystals (CsPbBr_2_Cl, NCs) loaded 3D printed NPG excited by 369 nm UV light, photos of the 3D printed the logo of Chinese Academy of Science and honeycomb with different luminous center. Schematic (left) and microscope images (right) of water droplets on e) unmodified and f) modified nanoporous glass surfaces. The lines and angles drawn on these images are for reference only.

To evaluate the transparency of NPG, the UV–vis transmission rates of four different components of the printed glass were measured. NPG prepared using DLP printing of different compositions shows different transparencies, as presented in Figure S8 (Supporting Information). The transparency of Al_2_O_3_‐SiO_2_ NPG is the highest, which is about 96% (**Figure** **5**b). The absorption cutoff edges of all samples were below 300 nm, except TiO_2_‐Al_2_O_3_‐SiO_2_ NPG. This is owing to the amorphous titanium dioxide in TiO_2_‐Al_2_O_3_‐SiO_2_ NPG would adjust the refractive index and dispersion of the glass, thus changing the UV cutoff edge.^[^ ^9^ ^]^ However, the transmittance of ZnO‐Al_2_O_3_‐SiO_2_ and CaO‐P_2_O_5_‐Al_2_O_3_‐ SiO_2_ is 86% and 69%, respectively, because Zn and Ca are glass network modifiers that may slightly aggregate in the glass network structure.^[^ ^58^ ^]^ In addition, EDS reveals the slightly segregation of Ca^2+^ cations in CaO‐P_2_O_5_‐Al_2_O_3_‐SiO_2_, which reduced the transparency.

Recently, 3D printing of rare earth luminescent ceramic micro‐nanostructures has been reported.^[^ ^59^ ^]^ However, researchers still use high‐temperature sintering to allow rare earth ions to enter the lattice structure to prepare light‐emitting devices, which is a common rare earth doping method.^[^ ^60^ ^]^ The transparency of the device is not compatible with the luminescent performance, and high‐temperature sintering also limits the selection of luminescent centers. For the simple and versatile photo‐functionalization of 3D printed NPG objects, we use rare earth ions (Eu^3+^, Tb^3+^), laser dyes (Rhodamine 6G) and luminescent nanocrystals (CsPbBr_2_Cl, NCs) to load into the nanoporous structure by soaking. The logo of the Chinese Academy of Sciences is printed with Al_2_O_3_‐SiO_2_ ink and calcined, and then the functional molecules are loaded by adsorption (Figure 5a). It was immersed in 0.5 mol L^−1^ aqueous solution of Tb(NO_3_)_3_ and Eu(NO_3_)_3_, 0.01 mol L^−1^ EtOH solution of Rhodamine 6G, and 0.1 mol L^−1^ toluene solution of quantum dots for 24 h, then the soaked glass is dried in an 80 °C oven for 2 h. All the luminescent species are successfully immersed into the nanopores of the glass, and the luminescence of Tb^3+^, Eu^3+^, Rhodamine 6G, and CsPbBr_2_Cl NCs can be observed under the excitation of a 396 nm lamp. In the photoluminescence spectrum, the emission peak of Eu^3+^ at 612 nm dominates the luminescence of NPG, while Tb^3+^ is the emission peak at 550 nm, and the photoluminescence peaks of Rhodamine 6G and CsPbBr_2_Cl NCs are located at 605 and 516 nm respectively (Figure 5c). The luminescent 3D‐printed logos and honeycombs under 369 nm UV excitation were demonstrated in Figure 5d. The high stability of dye molecules and nanocrystals inside nanoporous glass has been revealed in our previous work, owing to the size match between the pore and guest moeities.^[^ ^61^, ^62^ ^]^ However, the smaller rare earth ions loaded in the nanopores are easy to diffuse into the solution after soaking. Therefore, a sintering step is adapted for rare earth ions to enhance the stability of photo‐functionalization. The PL spectra of sintered sample was shown in Figure S9 (Supporting Information). The similar PL intensity of the samples after soaking indicates the high stability of rare earth ions in nanoporous glass after sintering, which results in the bonding of rare earth ions with glass network.^[^ ^63^ ^]^

The high surface area of NPG also enables surface state manipulation. The hydrophobicity‐modifying molecules were loaded into the pore structure, to realize the transformation of the hydrophilic NPG object into a hydrophobic one. To reduce the effect of surface modification on transparency, trimethylchlorosilane vapor loading was used to modify the surface of NPG.^[^ ^46^ ^]^ The measurement of the water droplet contact angle was used to demonstrate the hydrophobicity of the NPG surface modification. The surface contact angle of the unmodified NPG is 11.92° (Figure 5e). After loading TMCS molecules, the contact angle increases to 133.53° (Figure 5f), which demonstrated the successful hydrophobic modification of the surface of NPG by TMCS molecules.

## 3 Conclusion

In summary, 3D printing of multi‐component transparent nanoporous glass by DLP technique is presented, based on the sol–gel compositions, with the addition of photopolymerizing monomer and photoinitiator. This work develops an organic–inorganic hybrid oligomer ink for the DLP 3D printing of transparent nanoporous Al‐Si glass objects, which is a unique platform for fabricating multi‐component glasses objects with complex geometries and inherent porosity, such as Al_2_O_3_‐SiO_2_, ZnO‐Al_2_O_3_‐SiO_2_, TiO_2_‐Al_2_O_3_‐SiO_2_, CaO‐P_2_O_5_‐Al_2_O_3_‐SiO_2_. The photoluminescence and surface hydrophobicity modification are achieved by loading functional molecules into nanopores. The complex geometric nanoporous glass objects with a high specific surface area (> 496 m^2^ g^−1^) were fabricated by 3D printing followed by heat treatment. In contrast to the classical phase separation route, objects printed with the hybrid oligomer ink can obtain nanoporous glass at low temperatures, which avoids common and cumbersome processes such as high‐temperature melting and dangerous pickling corrosion of glass. Based on the well‐established sol‐gel chemistry, this work successfully extended the 3D printed transparent nanoporous glass from single component to multi‐components system. Overall, we achieved high print quality, complexity, transparency and rich chemical diversity of 3D printing nanoporous glass that was previously only realizable in dense glass, which will open prodigious opportunities in the functional design of nanoporous glass optical devices by loading functional molecules or nanoparticles into the nanoporous structure.

## 4 Experimental Section

### Materials

Aluminum L‐lactate (L‐Al), Calcium L‐lactate pentahydrate (L‐Ca), and Diphenyl (2,4,6‐trimethyibenzoyl) phosphine oxide (TPO) were purchased from Sigma‐Aldrich, USA. 3‐(Trimethoxysilyl) propyl Acrylate (APTMS), Tetraethyl orthosilicate (TEOS), Europium nitrate hexahydrate (Eu(NO_3_)_3_), Terbium nitrate hexahydrate (Tb(NO_3_)_3_), Chlorotrimethylsilane (TMCS), and dichloromethane were purchased from Aladdin, Shanghai, China. Titanium butoxide (TBT) and zinc acetate dihydrate (ZnAc) were purchased from Macklin, Shanghai, China. Ethanol (EtOH) was purchased from Qiangsheng Inc. Jiang Su, China. Ammonium dihydrogen phosphate (NH_4_H_2_PO_4_) was purchased from Richjoint, Shanghai, China.

### Sol Ink Preparation

The sol–gel ink was prepared as follows. First, prepare M1 consisting of L‐Al, L‐Ca, ZnAc, NH_4_H_2_PO_4_ and deionized water. L‐Al, L‐Ca, ZnAc, and NH_4_H_2_PO_4_ were weighed in proportion and dissolved in deionized water, then stir with a magnetic stirrer for 30 min. Second, a mixture of TEOS, TBT, and EtOH (denoted M2) was added to the M1 during the stirring process by slowly adding dropwise, and stirring the mixture until TEOS was fully reactive. Then, the M3 composed of APTMS and TPO was added. The solution was sealed in a dark vessel and stirred for ≈40 min until the formed sol could form a gel matrix upon exposure to UV radiation with a wavelength of 405 nm. The weight percentage L‐Al:TEOS:H_2_O:EtOH:APTMS:TPO at all stages of the process is presented in **Table** **2**. The four different components of sol‐gel ink correspond to different *X*_n_ and *Y*_n_ values, such as Al_2_O_3_‐SiO_2_ ink (*X*_1_ *= X*_2_ *= Y*_1_ *= Y*_2_ *=* 0), Al_2_O_3_‐ZnO‐SiO_2_ ink (*Y*_1_ *=* 0.47%; *X*_1_ *= X*_2_ *= Y*_2_ *=* 0), Al‐Ti‐Si ink (*Y*_2_ *=* 0.73%; *X*_1_ *= X_2_* *= Y*_1_ *=* 0), Al_2_O_3_‐P_2_O_5_‐CaO‐SiO_2_ ink (*X*_1_ *=* 0.66%, *X*_2_ *=* 0.25%; *Y*_1_ *=* *Y*_2_ *=* 0)

**Table 2:** Sol–gel ink composition.

M1 [wt%]					M2 [wt%]			M3 [wt%]	
L‐Al	L‐Ca	NH_4_H_2_PO_4_	ZnAc	H_2_O	TEOS	TBT	EtOH	APTMS	TPO
6.30‐Σ*X*_n_‐*Y*_n_	*X*_1_	*X*_2_	*Y*_1_	15.97	44.61	*Y*_2_	27.94	4.94	0.25

### Sample Fabrication

The 3D models of a sample are designed by AutoCAD (Autodesk, California, USA), and printed using a DLP 3D printer (MaxUV, Asiga). The printing parameters include using a UV‐LED light source (405 nm) with a light intensity of 15 mW cm^−2^, and the exposure time was set to be 6 s for a layer thickness of 100 µm. After printing, the structures were placed in a closed vessel with a tiny hole and aging, drying in a 70 °C oven for 7 days. Then, dried samples were sintered at 700 °C (temperature regime, 1 °C min^−1^ to 400 °C for 120 min, 1 °C min^−1^ to 700 °C for 120 min) in a muffle furnace.

Glass discs are used to test the transmittance. Their preparation process is that the ink is casted into the transparent cylinder mold (inner diameter: ϕ 10 mm × 4 mm) and exposing it to UV‐LED light (405 nm) for 60 s. After sintering processing, glass disks with a diameter of about 5 mm and a thickness of about 1.5 mm is obtained.

For the loading of functionalized particle, the four transparent nanoporous glass samples respectively were immersed in 0.5 mol L^−1^ aqueous solution of Tb(NO_3_)_3_ and Eu(NO_3_)_3_, 0.01 mol L^−1^ EtOH solution of Rhodamine 6G, and 0.1 mol L^−1^ toluene solution of CsPbBr_2_Cl for 24 h. Then the samples immersed in rare earth ions, dye molecules and nanocrystals were rinsed with deionized water, ethanol and toluene, respectively. Finally, the soaked all glass samples were placed in an oven at 80 °C for 2 h.

The nanoporous glass was placed in a 100 °C oven for 12 h to eliminate water. Next, a solution of TMCS (1 mL) in dichloromethane (3 mL) was prepared. Dried glass and solution (2 mL) were transferred to a vacuum desiccator, then it was sealed and heated to 50 °C for 100 h. After modification time, the temperature was raised to 70 °C and continued to evacuate to remove residual gas, followed by natural cooling to obtain hydrophobically modified transparent nanoporous glasses.

### Materials Characterization

Shrinkage was measured by micrometer and analytical balances for printed cuboids with a size of 10 × 15 × 3 mm^3^, after printing, drying, and sintering. TG‐DTA analyses of powders ground dry sample were performed with an EXSTAR TG/DTA7300 (SII, Japan) in the temperature range of 50–1000 °C, at a heating rate of 10 °C min^−1^. Field‐emission scanning electron microscopy (FE‐SEM; Auriga S40, Zeiss, Oberkochen, Germany). TEM images of the nanoporous glass were obtained using a TEM (HEOL‐2010). The samples were previously dropped on clean bare wafers and later transfer onto a 300‐mesh copper TEM grid by spot cleaning. A Link‐Isis (Oxford Instruments) energy dispersive X‐Ray spectroscopy (EDX) detector was used to determine and map chemical compositions. X‐ray diffractometer (XRD, PANalytical X′Pert Pro, the Netherlands) with CuK_α1_ radiation in the 2*θ* range of 10–90° was used to characterize the morphology, composition, and glass structure of the 3D printing structure after sintering.

The specific surface area and pore size distribution were measured with the use of an N_2_ adsorption‐desorption apparatus (Autosorb iQ, Anton Paar, Austria) at −196 °C. All samples were degassed under vacuum at 150 °C for 10 h before analysis. The surface area and the pore size distribution were respectively calculated by Brunauer–Emmett–Teller (BET) and Barrett–Joyner–Halenda (BJH) equations, over acquired adsorption data in the P/P_0_ range 0–1. The transmittance spectra were evaluated in the ranges of 200−900 nm using a UV–vis spectrophotometer (LAMBDA 750, PerkinElmer, USA). Photoluminescence (PL) was obtained using the instrument FLS1000 (Edinburgh Instruments Ltd.), with a pump wavelength of 369 nm. Place a drop of 10 µL of deionized water on the nanoporous glass surface at 20 °C. The contact angle was recorded using a Ramé–Hart model 100 contact angle goniometers. The linear shrinkage is defined as *S* = – (Δ*L*/*L*_0_) × 100%, Δ*L* = *L* – *L*_0_, where *L*_0_ is the original length of samples, and *L* is the length of the sample in three directions after each heat treatment. The anisotropy factor (*K*) can be introduced to evaluate its influence on the final dimensional precision of the nanoporous glass, which was calculated by *K*_xy_ = 100(1 – *S*_x_ */S*_y_).

## Conflict of Interest

The authors declare no conflict of interest.

## Supporting information

Supporting Information

## Data Availability

The data that support the findings of this study are available from the corresponding author upon reasonable request.

## References

[1] B. J. Scott , G. Wirnsberger , G. D. Stucky , Chem. Mater. 2001, 13, 3140.

[2] M. Hartmann , Chem. Mater. 2005, 17, 4577.

[3] T. Wu , Z. Wu , Y. He , Z. Zhu , L. Wang , K. Yin , Chin. Opt. Lett. 2022, 20, 033801.

[4] J. Klein , M. Stern , G. Franchin , M. Kayser , C. Inamura , S. Dave , J. C. Weaver , P. Houk , P. Colombo , M. Yang , N. Oxman , 3D Print. Addit. Manuf. 2015, 2, 92.

[5] E. Baudet , Y. Ledemi , P. Larochelle , S. Morency , Y. Messaddeq , Opt. Mater. Express 2019, 9, 2307.

[6] I. Cooperstein , E. Shukrun , O. Press , A. Kamyshny , S. Magdassi , ACS Appl. Mater. Interfaces 2018, 10, 18879. 29741081 10.1021/acsami.8b03766

[7] F. Kotz , K. Arnold , W. Bauer , D. Schild , N. Keller , K. Sachsenheimer , T. M. Nargang , C. Richter , D. Helmer , B. E. Rapp , Nature 2017, 544, 337. 28425999 10.1038/nature22061

[8] S. N. Grigoriev , R. S. Khmyrov , M. A. Gridnev , T. V. Tarasova , A. V. Gusarov , J. Manuf. Sci. Eng. 2022, 144, 061001.

[9] J. F. Destino , N. A. Dudukovic , M. A. Johnson , D. T. Nguyen , T. D. Yee , G. C. Egan , A. M. Sawvel , W. A. Steele , T. F. Baumann , E. B. Duoss , T. Suratwala , R. Dylla‐Spears , Adv. Mater. Technol. 2018, 3, 1700323.

[10] K. Sasan , A. Lange , T. D. Yee , N. Dudukovic , D. T. Nguyen , M. A. Johnson , O. D. Herrera , J. H. Yoo , A. M. Sawvel , M. E. Ellis , C. M. Mah , R. Ryerson , L. L. Wong , T. Suratwala , J. F. Destino , R. Dylla‐Spears , ACS Appl. Mater. Interfaces 2020, 12, 6736. 31934741 10.1021/acsami.9b21136

[11] J. Ha , K. Sasan , T. D. Yee , A. P. Lange , D. T. Nguyen , N. Dudukovic , R. Dylla‐Spears , Adv. Photonics Res. 2022, 3, 2200017.

[12] R. Dylla‐Spears , T. D. Yee , K. Sasan , D. Nguyen , N. A. Dudukovic , J. M. Ortega , M. A. Johnson , O. D. Herrera , F. J. Ryerson , L. L. Wong , Sci. Adv. 2020, 6, 7429. 10.1126/sciadv.abc7429 PMC7673801 33208366

[13] G. Balčas , M. Malinauskas , M. Farsari , S. Juodkazis , Adv. Funct. Mater. 2023, 33, 2215230.

[14] D. Gonzalez‐Hernandez , S. Varapnickas , G. Merkininkaitė , A. Čiburys , D. Gailevičius , S. Šakirzanovas , S. Juodkazis , M. Malinauskas , Photonics 2021, 8, 577.

[15] M.‐Q. Cai , Q. Wang , C.‐H. Tu , Y.‐N. Li , H.‐T. Wang , Chin. Opt. Lett. 2022, 20, 010502.

[16] J. Bauer , C. Crook , T. Baldacchini , Science 2023, 380, 960. 37262172 10.1126/science.abq3037

[17] X. Wen , B. Zhang , W. Wang , F. Ye , S. Yue , H. Guo , G. Gao , Y. Zhao , Q. Fang , C. Nguyen , X. Zhang , J. Bao , J. T. Robinson , P. M. Ajayan , J. Lou , Nat. Mater. 2021, 20, 1506. 34650230 10.1038/s41563-021-01111-2

[18] C. Xin , Z. Li , L. Hao , Y. Li , Mater. Des. 2023, 227, 111736.

[19] D. G. Moore , L. Barbera , K. Masania , A. R. Studart , Nat. Mater. 2020, 19, 212. 31712744 10.1038/s41563-019-0525-y

[20] J. Wang , B. Zheng , P. Wang , J. Non‐Cryst. Solids 2020, 550, 120362.

[21] D. Cheng , C. Wei , Y. Huang , Z. Zhang , D. Wang , Z. Liu , M. Newman , T. Ma , Y.‐H. Chueh , X. Zhang , Z. Liu , L. Li , Addit. Manuf. 2022, 49, 102481.

[22] E. Shukrun , I. Cooperstein , S. Magdassi , Adv. Sci. 2018, 5, 1800061. 10.1002/advs.201800061 PMC6096996 30128232

[23] R. Gvishi , I. Sokolov , J. Sol‐Gel Sci. Technol. 2020, 95, 635.

[24] L. Duan , C. Wang , W. Zhang , B. Ma , Y. Deng , W. Li , D. Zhao , Chem. Rev. 2021, 121, 14349. 34609850 10.1021/acs.chemrev.1c00236

[25] J. C. Chen , J. Li , B. N. Li , Z. C. Chen , X. Bai , L. Zhang , J. He , J. Non‐Cryst. Solids 2023, 613, 122329.

[26] Z. Hu , Y. Jiang , F. Zhou , C. Chen , J. He , Z. Zhan , Z. Liu , J. Du , L. Zhang , Y. Leng , Adv. Opt. Mater. 2023, 11, 2202131.

[27] Y. B. Chu , Y. Yang , L. Liao , Y. G. Liu , Y. X. Ma , X. W. Hu , Y. B. Wang , Y. B. Xing , J. G. Peng , H. Q. Li , N. L. Dai , J. Y. Li , L. Y. Yang , ACS Photonics 2018, 5, 4014.

[28] I. A. Aksay , D. M. Dabbs , M. Sarikaya , J. Am. Ceram. Soc. 1991, 74, 2343.

[29] D. Enke , F. Janowski , W. Schwieger , Microporous Mesoporous Mater. 2003, 60, 19.

[30] J. J. Ren , L. Zhang , H. Eckert , J. Sol‐Gel Sci. Technol. 2014, 70, 482.

[31] E. Shukrun Farrell , Y. Schilt , M. Y. Moshkovitz , Y. Levi‐Kalisman , U. Raviv , S. Magdassi , Nano Lett. 2020, 20, 6598. 32787154 10.1021/acs.nanolett.0c02364 PMC7496731

[32] Y. Zhang , L. Xia , D. Zhai , M. Shi , Y. Luo , C. Feng , B. Fang , J. Yin , J. Chang , C. Wu , Nanoscale 2015, 7, 19207. 26525451 10.1039/c5nr05421d

[33] E. S. Farrell , N. Ganonyan , I. Cooperstein , M. Y. Moshkovitz , Y. Amouyal , D. Avnir , S. Magdassi , Appl. Mater. Today 2021, 24, 101083.

[34] Z. Dong , H. Cui , H. Zhang , F. Wang , X. Zhan , F. Mayer , B. Nestler , M. Wegener , P. A. Levkin , Nat. Commun. 2021, 12, 10.1038/s41467-020-20498-1. PMC7801408 33431911

[35] E. Shukrun Farrell , R. Siam , M. Y. Moshkovitz , D. Avnir , R. Abu‐Reziq , S. Magdassi , Addit. Manuf. 2022, 60, 103265.

[36] L. Zhang , A. Bogershausen , H. Eckert , J. Am. Ceram. Soc. 2005, 88, 897.

[37] J. He , P. F. Ma , G. Zhang , R. H. Li , L. Zhang , RSC Adv. 2016, 6, 99149.

[38] C. C. de Araujo , L. Zhang , H. Eckert , J. Mater. Chem. 2006, 16, 1323.

[39] G. Merkininkaitė , E. Aleksandravičius , M. Malinauskas , D. Gailevičius , S. Šakirzanovas , Opto‐Electron. Adv. 2022, 5, 210077.

[40] G. Grenci , G. D. Giustina , A. Pozzato , E. Zanchetta , M. Tormen , G. Brusatin , Microelectron. Eng. 2012, 98, 134.

[41] J. Feng , H. Zhang , Chem. Soc. Rev. 2013, 42, 387. 22977887 10.1039/c2cs35069f

[42] W. Wang , Q. Chen , Y. Zhao , Y. Le , S. Ye , M. Wan , X. Huang , G. Dong , Chin. Opt. Lett. 2022, 20, 021603.

[43] R. R. Deshpande , H. Eckert , J. Mater. Chem. 2009, 19, 3419.

[44] C. J. Brinker , K. D. Keefer , D. W. Schaefer , C. S. Ashley , J. Non‐Cryst. Solids 1982, 48, 47.

[45] J. Ren , L. Zhang , H. Eckert , J. Phys. Chem. C 2014, 118, 4906.

[46] N. Ganonyan , G. Bar , R. Gvishi , D. Avnir , RSC Adv. 2021, 11, 7824. 35423309 10.1039/d1ra00671a PMC8695093

[47] C. J. Brinker , K. D. Keefer , D. W. Schaefer , R. A. Assink , B. D. Kay , C. S. Ashley , J. Non‐Cryst. Solids 1984, 63, 45.

[48] M. Yamane , S. Aso , T. Sakaino , J. Mater. Sci. 1978, 13, 865.

[49] B. Zagrajczuk , M. Dziadek , Z. Olejniczak , B. Sulikowski , K. Cholewa‐Kowalska , M. Laczka , J. Mol. Struct. 2018, 1167, 23.

[50] C. Lin , J. A. Ritter , M. D. Amiridis , J. Non‐Cryst. Solids 1997, 215, 146.

[51] A. B. Attygalle , A. Svatos , C. Wilcox , S. Voerman , Anal. Chem. 2002, 67, 1558. 10.1021/ac00082a016 8030784

[52] M. Bacci , Spectrochim. Acta, Part A 1972, 28, 2286.

[53] M. Rank , A. Sigel , Y. Bauckhage , S. Suresh‐Nair , M. Dohmen , C. Eder , C. Berge , A. Heinrich , in 3D Printing of Optical Components, Springer International Publishing, Springer, Cham, 2021, Chapter 3.

[54] J. D. Joannopoulos , P. R. Villeneuve , S. Fan , Nature 1997, 386, 143.

[55] M. Minkov , V. Savona , Sci. Rep. 2014, 4, 10.1038/srep05124. PMC4038819 24874589

[56] Y. Fu , X. Hu , Q. Gong , Phys. Lett. A 2013, 377, 329.

[57] S. Lin , C. Ionescu , K. J. Pike , M. E. Smith , J. R. Jones , J. Mater. Chem. 2009, 19, 1276.

[58] K. Xu , Q. Meng , L. Li , M. Zhu , Ceram. Int. 2021, 47, 18905.

[59] J. Winczewski , M. Herrera , C. Cabriel , I. Izeddin , S. Gabel , B. Merle , A. Susarrey Arce , H. Gardeniers , Adv. Opt. Mater. 2022, 10, 2102758.

[60] H. K. Nie , F. F. Wang , J. T. Liu , K. J. Yang , B. T. Zhang , J. L. He , Chin. Opt. Lett. 2021, 19, 091407.

[61] R. Li , L. Zhang , J. Ren , T. B. de Queiroz , A. S. S. de Camargo , H. Eckert , J. Non‐Cryst. Solids 2010, 356, 2089.

[62] Z. Bai , J. He , Y. Wang , K. Wang , R. Li , L. Zhang , J. Lumin. 2017, 192, 675.

[63] J. He , P. Ma , Y. Wang , R. Li , X. Yuan , L. Zhang , J. Non‐Cryst. Solids 2016, 431, 130.

