# Supplementary material for: Additive Manufacturing of Transparent Multi‐Component Nanoporous Glasses
Source: Adv Sci (Weinh). 2023 Oct 23;10(35):2305775. doi: 10.1002/advs.202305775 (PMC10724418; doi:10.1002/advs.202305775)
Supplement: Supplementary file 1 — Supporting Information [file ADVS-10-2305775-s001.pdf]

ADVANCED SCIENCE
Open Access

Supporting Information

for *Adv. Sci.*, DOI 10.1002/advs.202305775

Additive Manufacturing of Transparent Multi-Component Nanoporous Glasses

Beining Li, Zhenjiang Li, Ido Cooperstein, Wenze Shan, Shuaipeng Wang, Benxue Jiang, Long Zhang, Shlomo Magdassi* and Jin He**

Supplementary Information:

Additive Manufacturing of Transparent Multi-component Nanoporous Glasses

Beining Li, Zhenjiang Li, Ido Cooperstein, Wenze Shan, Shuaipeng Wang, Benxue Jiang, Long Zhang, Shlomo Magdassi*, Jin He**

B. Li, W. Shan, S. Wang, Benxue Jiang, L. Zhang, J. He
Key Laboratory of Materials for High Power Lasers
Shanghai Institute of Optics and Fine Mechanics, Chinese Academy of Sciences
Shanghai 201800, China
E-mail: jiangbx@siom.ac.cn(B. J.); jhe@siom.ac.cn (J. H.)
B. Li, Z. Li, W. Shan,
College of Materials Science and Opto-Electronic Technology
University of Chinese Academy of Sciences
Beijing 100083, China
Z. Li
Shanghai Institute of Applied Physics, Chinese Academy of Sciences
Shanghai 201800, China
I. Cooperstein, S. Magdassi, J. He
Casali Center of Applied Chemistry, Institute of Chemistry
The Hebrew University of Jerusalem
Jerusalem 9190401, Israel
E-mail: Magdassi@mail.huji.ac.il (S. M.); jhe@siom.ac.cn (J. H.)

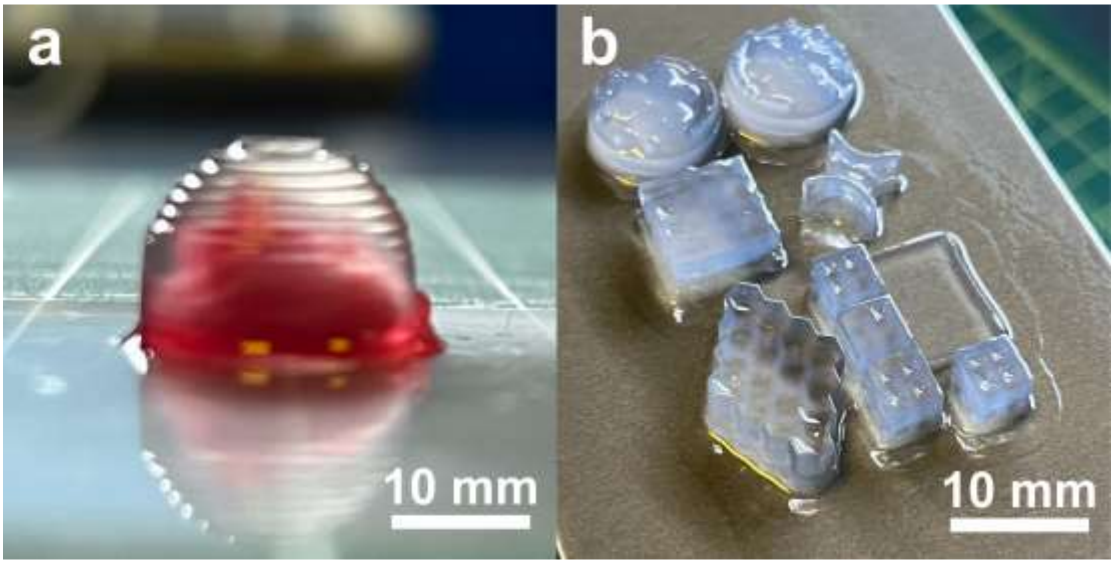

Figure S1. (a) The hollow hyper hemispheric gel (the red part is the dye injected into the cavity to enhance the display), (b) as well as other complex structures 3D printed.

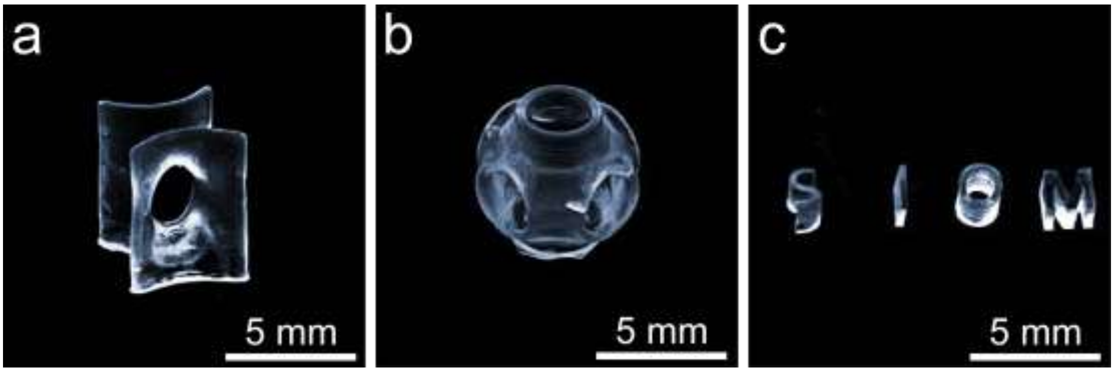

Figure S2. The centimeter-level structure photos of transparent NPG after sintering, such as (a, b) triply periodic minimal surface structure and (c) the letter "SIOM"

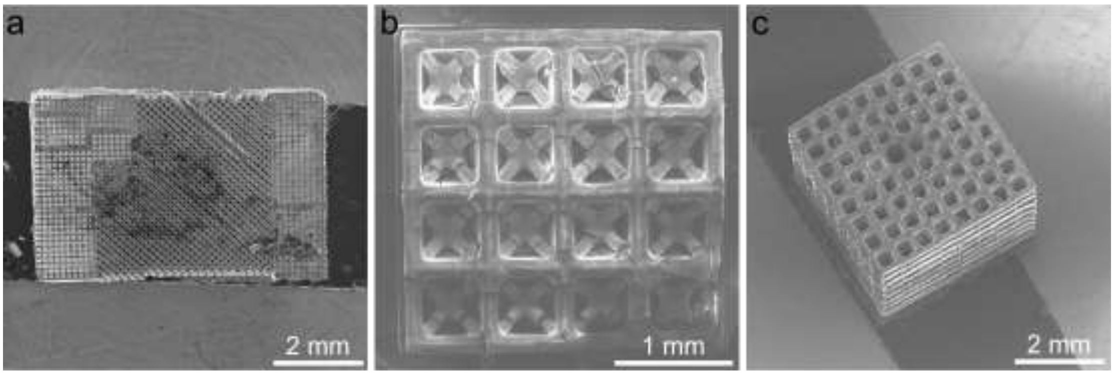

Figure S3. The millimeter-level structure SEM images of transparent NPG after sintering such as (a) photonic gate and (b, c) lattice.

During the gel to glass transition, capillary forces from the evaporation of the

solution and thermal decomposition of organic moieties possibly lead to shrinkage and cracks.

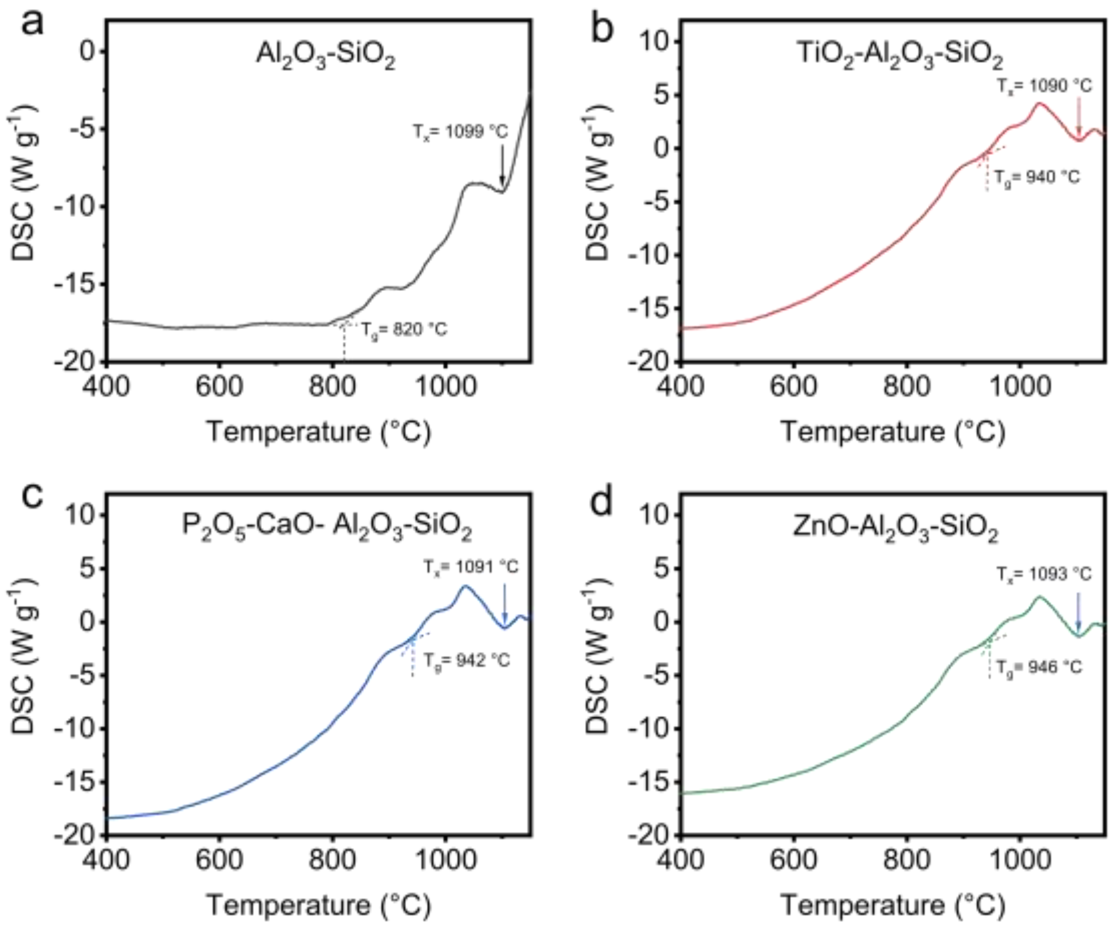

Figure S4 Differential scanning calorimetry of the 3D printed multi-component glass, (a) Al_2O_3-SiO_2, (b) TiO_2-Al_2O_3-SiO_2, (c) P_2O_5-CaO-Al_2O_3-SiO_2, and (d) ZnO-Al_2O_3-SiO_2.

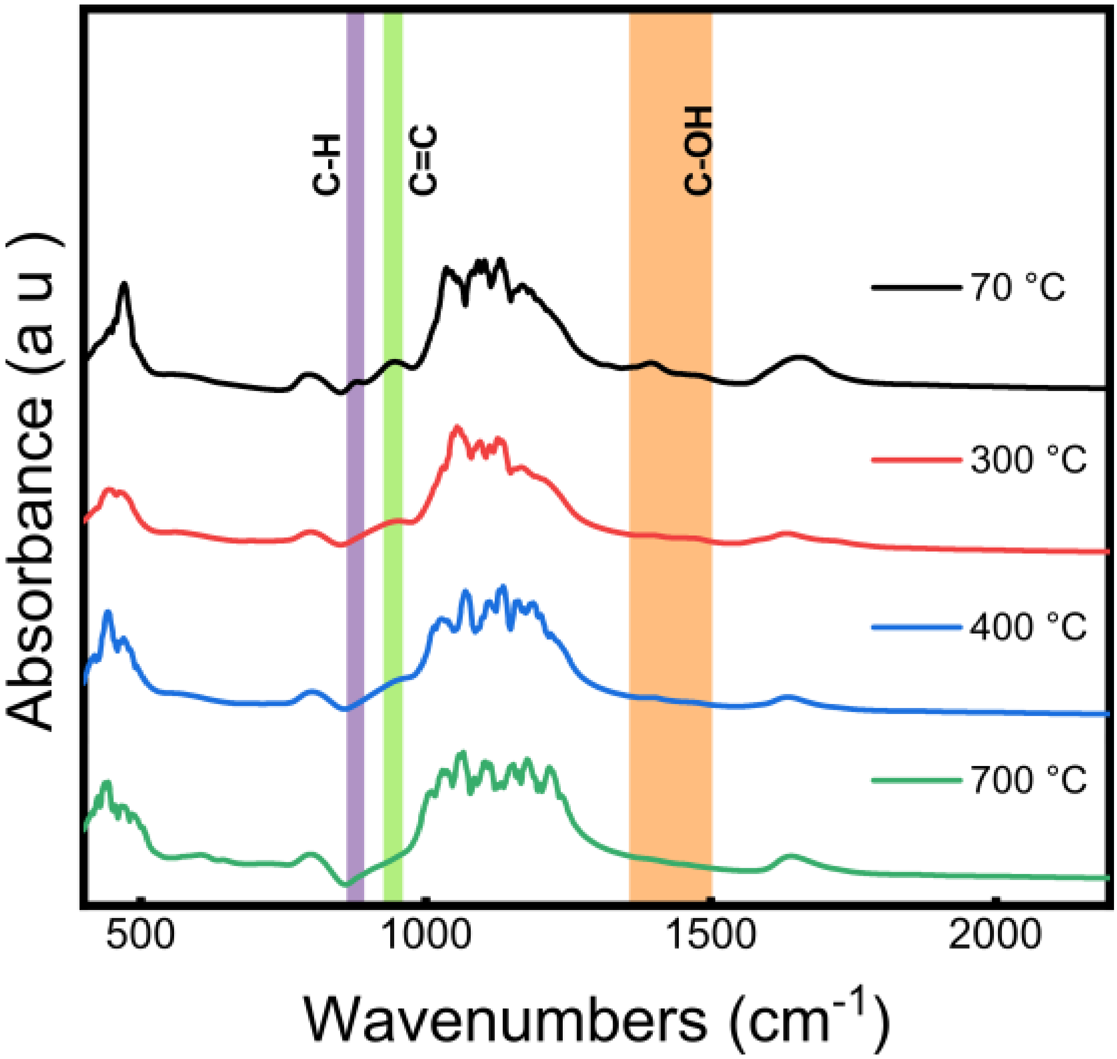

Figure S5 Fourier transform infrared (FTIR) spectra of xerogels treated at different temperatures. The xerogels were dried at 70 °C (black curve) and subsequently sintered at 300 °C (red curve), 400°C (blue curve) and 700°C (green curve) for 2 h.

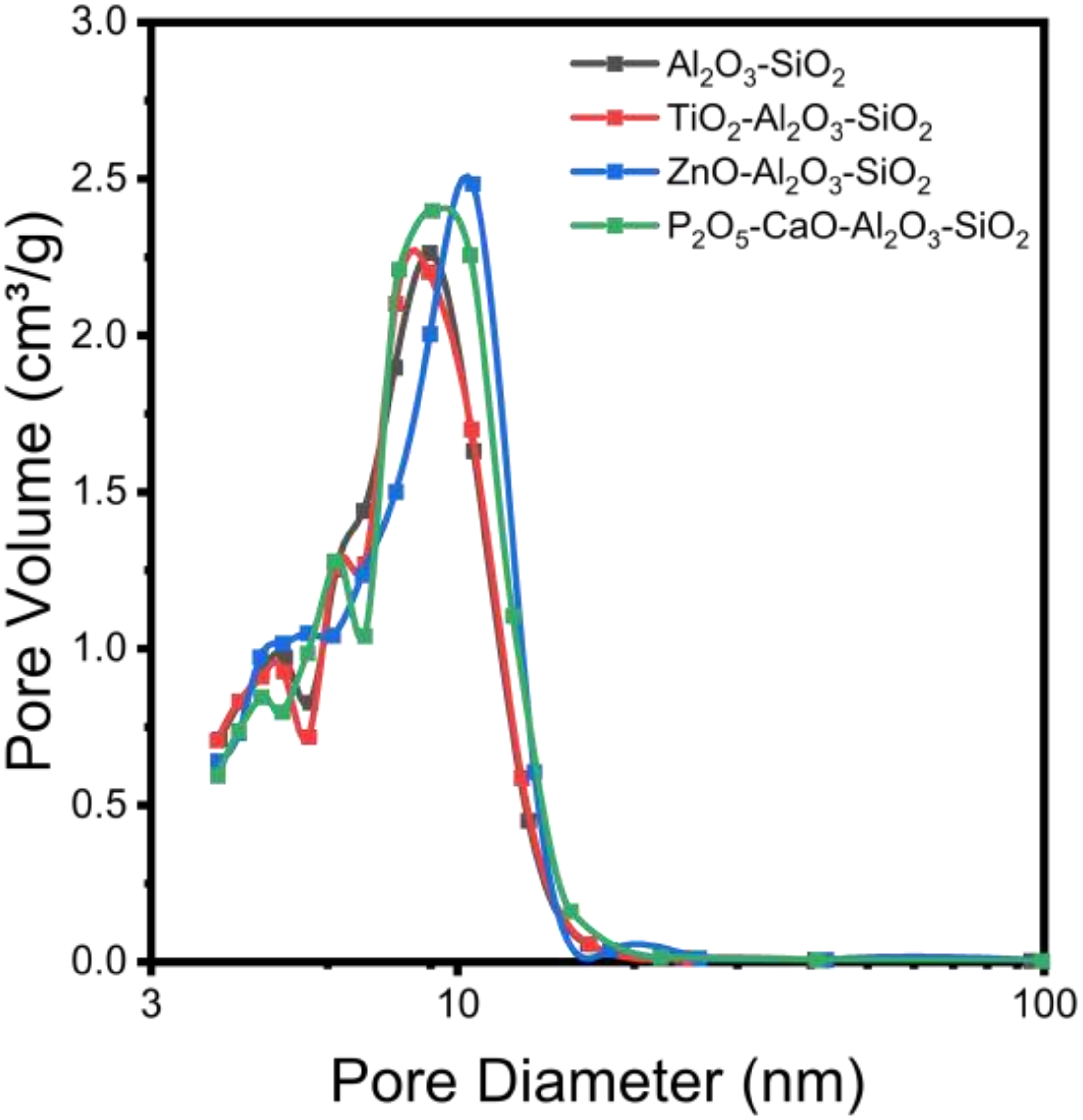

Figure S6 Pore volume distribution of the glasses sintered at 700 °C, Al_2O_3-SiO_2 (red), TiO_2-Al_2O_3-SiO_2 (blue), ZnO_2- Al_2O_3-SiO_2 (green), and P_2O_5-CaO- Al_2O_3-SiO_2 (purple) samples.

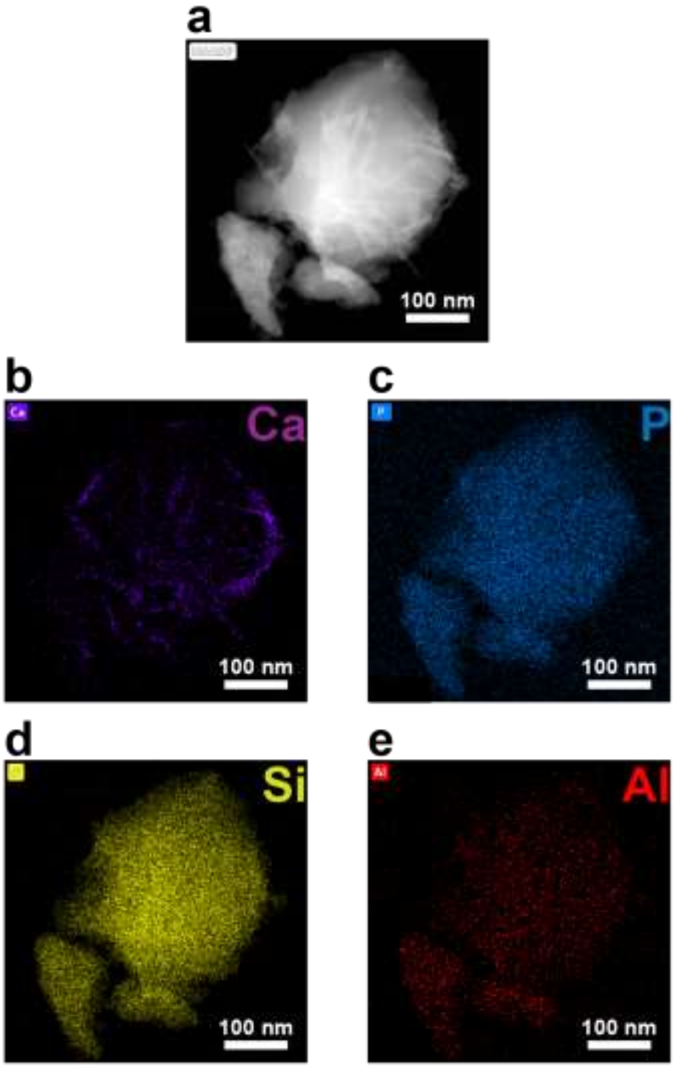

Figure S7. (a) TEM images and EDX spectrum of Al-P-Ca-Si transparent nonporous glasses and elemental mapping for (b) Ca, (c) P, (d) Si, (e) Al showing the composition of the glass.

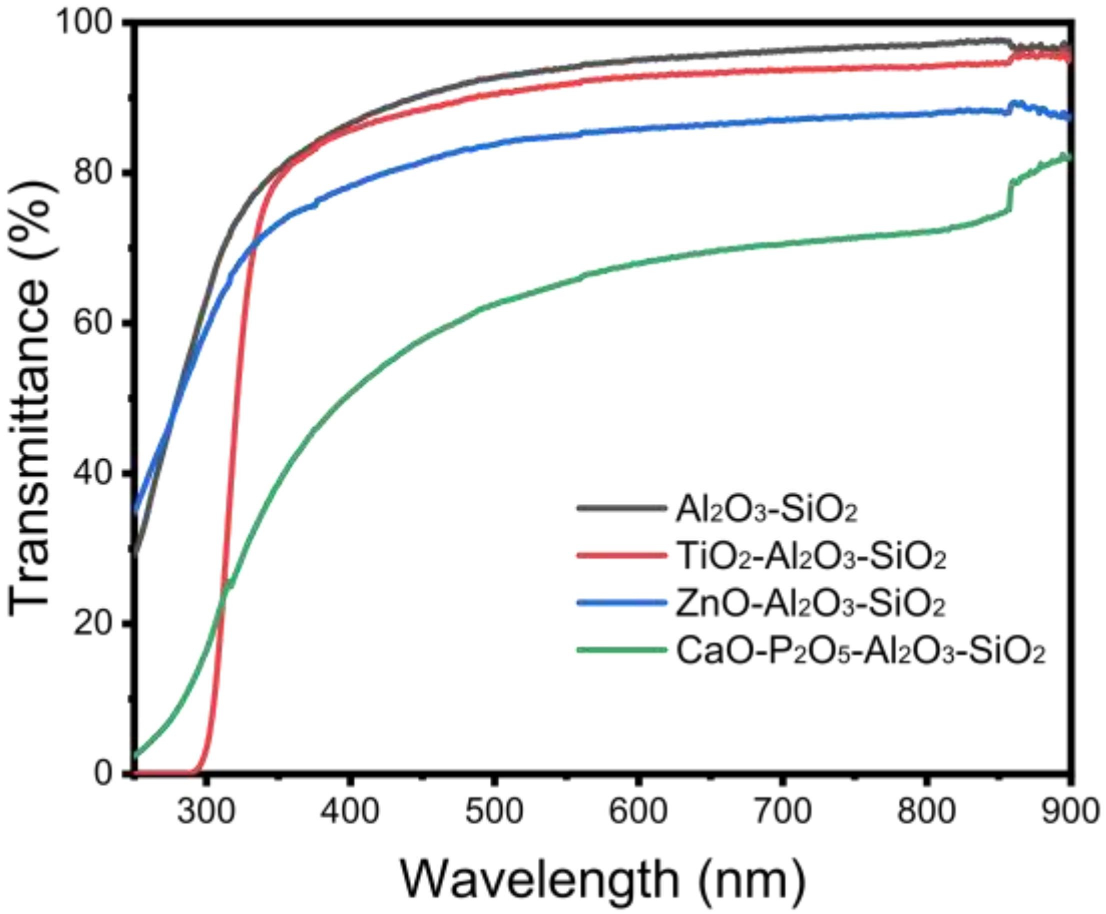

Figure S8. UV–vis optical transmission spectra of 3D printed nanoporous glasses with different compositions.

The stability experiments of rare earth ion loaded nanopore glass are as follows: First, take five transparent nanoporous glasses and name them G1, G2, G3, SG1, SG2 respectively. Then all glasses were soaked in a 1 mol L^{-1} $Eu(NO)_3$ solution for 24 hours, then the surface was rinsed with deionized water and placed in an oven at 80°C for 12 hours. Next, G2 and G3 was soaked in deionized water for 1 h and 6h, respectively. SG1 and SG2 was sintered at 500 °C for 6 h, then SG2 was soaked in deionized water for 6 h. The treated G2, G3 and SG2 samples were washed with deionized water and baked in an oven at 80 °C for 12 hours.

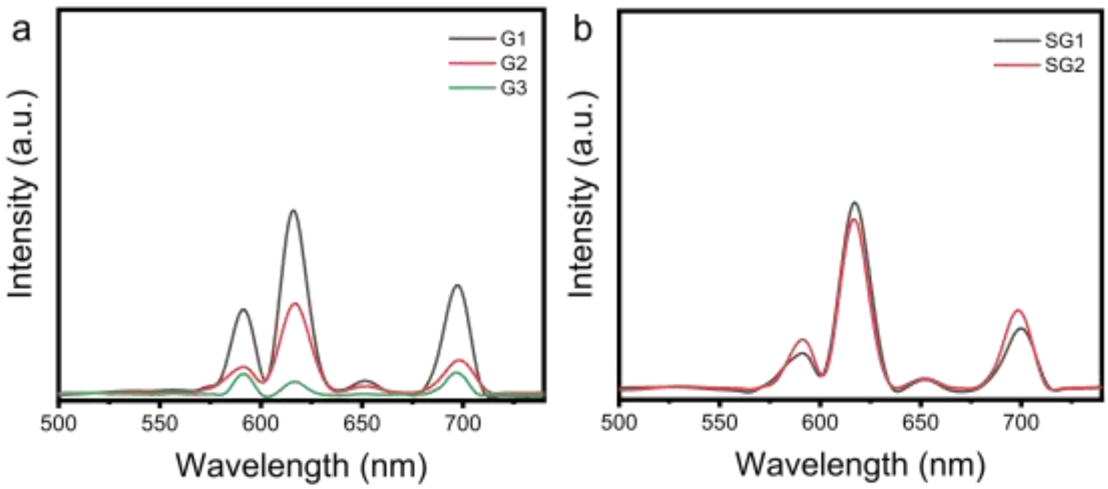

Figure S9 (a)PL spectra of transparent NPG loaded with Eu3+ when immersed in deionized water for 0, 1,6 h. (b)PL spectra of transparent NPG loaded with Eu3+ before and after being immersed in deionized water after being sintered at 500 °C.
